# Construction and validation of the risk assessment scale for deep vein thrombosis in high altitude plateau areas

**DOI:** 10.3389/fmed.2026.1742559

**Published:** 2026-03-11

**Authors:** Xiaolin Sun, Shiqin Pan, Mingqin Luo, Yuemei Li, Hongmei Ma, Lijuan Sun, Yincong Luo, Yaqian Tong

**Affiliations:** 1 Department of Critical Care Medicine, Qinghai Provincial People's Hospital, Xining, China; 2 Nursing Department, Qinghai Provincial People's Hospital, Xining, China; 3 School of Clinical Medicine, Qinghai University, Xining, China

**Keywords:** deep vein thrombosis, plateau, reliability and validity, risk, scale

## Abstract

**Objective:**

To develop a deep vein thrombosis (DVT) risk assessment scale suitable in plateau areas and to test its reliability and validity.

**Methods:**

Based on a previous large-scale survey, combined with literature search and group discussions, an item pool for the DVT risk assessment scale in high-altitude areas was constructed. The items were discussed and revised through two rounds of Delphi expert consultations. The Analytic Hierarchy Process was used to determine the weight of each item and assign scores. A convenience sampling method was employed to select 214 patients admitted to a tertiary Hospital in Qinghai People from October 2023 to December 2023 as the research subjects. Discriminant validity and risk stratification were used to classify the overall risk level of the scale.

**Results:**

The Risk Assessment Scale for Deep Vein Thrombosis in Plateau Areas consists of 5 dimensions and 31 items. The Cronbach’s α coefficient was 0.953, and the split-half coefficient was 0.957. The scale-level content validity index was 0.91, and the item-level content validity index ranged from 0.72 to 1.00. The area under the ROC curve was 0.73, with a 95% CI (0.655, 0.805), and the cutoff value was 14.5 points.

**Conclusion:**

The Risk Assessment Scale for Deep Vein Thrombosis in Plateau Areas developed in this study has good reliability and validity, and can scientifically and effectively assess the risk of DVT formation in hospitalized patients in high-altitude areas.

## 1 Introduction

Deep Vein Thrombosis (DVT) is a pathological condition characterized by abnormal blood clot formation within deep veins, leading to impaired venous return (1). As a component of venous thromboembolism (VTE), DVT represents the third most prevalent cardiovascular disorder, following myocardial infarction and stroke (2). Without prompt intervention, thrombus detachment may occur, resulting in life-threatening pulmonary embolism (PE) through systemic circulation (3). The nonspecific clinical manifestations and insidious onset of DVT contribute to its high rates of missed and misdiagnosis (4, 5). Consequently, accurate assessment and early identification are critical for effective prevention and clinical management of this condition.

Currently, internationally adopted DVT risk assessment tools include the Padua Prediction Score, Autar Scale, Caprini risk assessment scale, and Wells Score (6–8), all of which demonstrate good general applicability. However, these conventional scales show limited suitability for plateau regions due to their failure to account for unique environmental factors and the distinctive pathophysiological status of patients in high-altitude areas. Their sensitivity is significantly reduced in plateau settings, rendering them inadequate to effectively reflect and accurately evaluate the impact of high-altitude conditions on DVT risk. Epidemiological studies indicate that the incidence rate of DVT among hospitalized patients in high-altitude regions is 2.19-fold higher than that in plain areas (9). The distinctive geographical conditions of plateau environments induce compensatory polycythemia, increased blood viscosity, and a prothrombotic state (10). Moreover, chronic hypoxia triggers elevated inflammatory cytokines, dysregulation of vasoactive factors, and subsequent vascular endothelial dysfunction (11, 12)—all established risk factors for DVT pathogenesis. Therefore, the items in currently common DVT risk assessment scales fail to capture altitude-specific factors, such as the pathophysiological consequences of chronic hypoxia, compensatory polycythemia, and other unique risk factors prevalent in plateau populations. Our research team previously conducted large-scale DVT screening among plateau-region inpatients (13), identifying altitude-specific risk determinants. Building upon these findings, this study developed a tailored risk assessment scale through systematic literature review and Delphi expert consultation, followed by rigorous reliability and validity testing. The proposed instrument aims to enhance risk stratification sensitivity and provide clinically actionable guidance for early DVT detection in high-altitude populations.

## 2 Methods

### 2.1 Establishment of the research team

The research team consisted of 15 members, including 3 nursing department directors, 2 division head nurses, 5 ward nurse managers, 4 clinical nursing specialists, and 1 graduate research assistant. Among them, there were 6 with senior professional titles, 8 with intermediate titles, 12 with bachelor’s degrees, 2 with master’s degrees, and 1 graduate student.

### 2.2 Theoretical basis

This study was based on the thrombosis triad theory proposed by the renowned German physician Rudolf Virchow (14). The theory elucidates the pathophysiological basis of thrombosis and summarizes three key elements: hypercoagulability, hemodynamic abnormalities, and vascular endothelial injury. This theory not only reveals the underlying mechanisms of thrombosis but also identifies major risk factors, providing crucial theoretical guidance for clinical prevention and management of thrombosis. Based on this theory, we established the initial item pool for the scale.

### 2.3 Preliminary development of the scale item pool

#### 2.3.1 Database search

Chinese databases included Wanfang and CNKI; English databases included PubMed and Web of Science.

#### 2.3.2 Search terms

Chinese/English search terms: high-altitude/plateau, deep vein thrombosis/venous thromboembolism, risk factors/factors influencing, assessment tool/risk assessment/assessment scales.

#### 2.3.3 Literature inclusion and exclusion criteria

Inclusion criteria: studies on DVT assessment systems; studies on DVT influencing/risk factors.

Exclusion criteria: interventional studies; conference papers; theses; abstracts; duplicate publications; priority was given to English literature.

#### 2.3.4 Initial scale item pool

**Figure 1:**
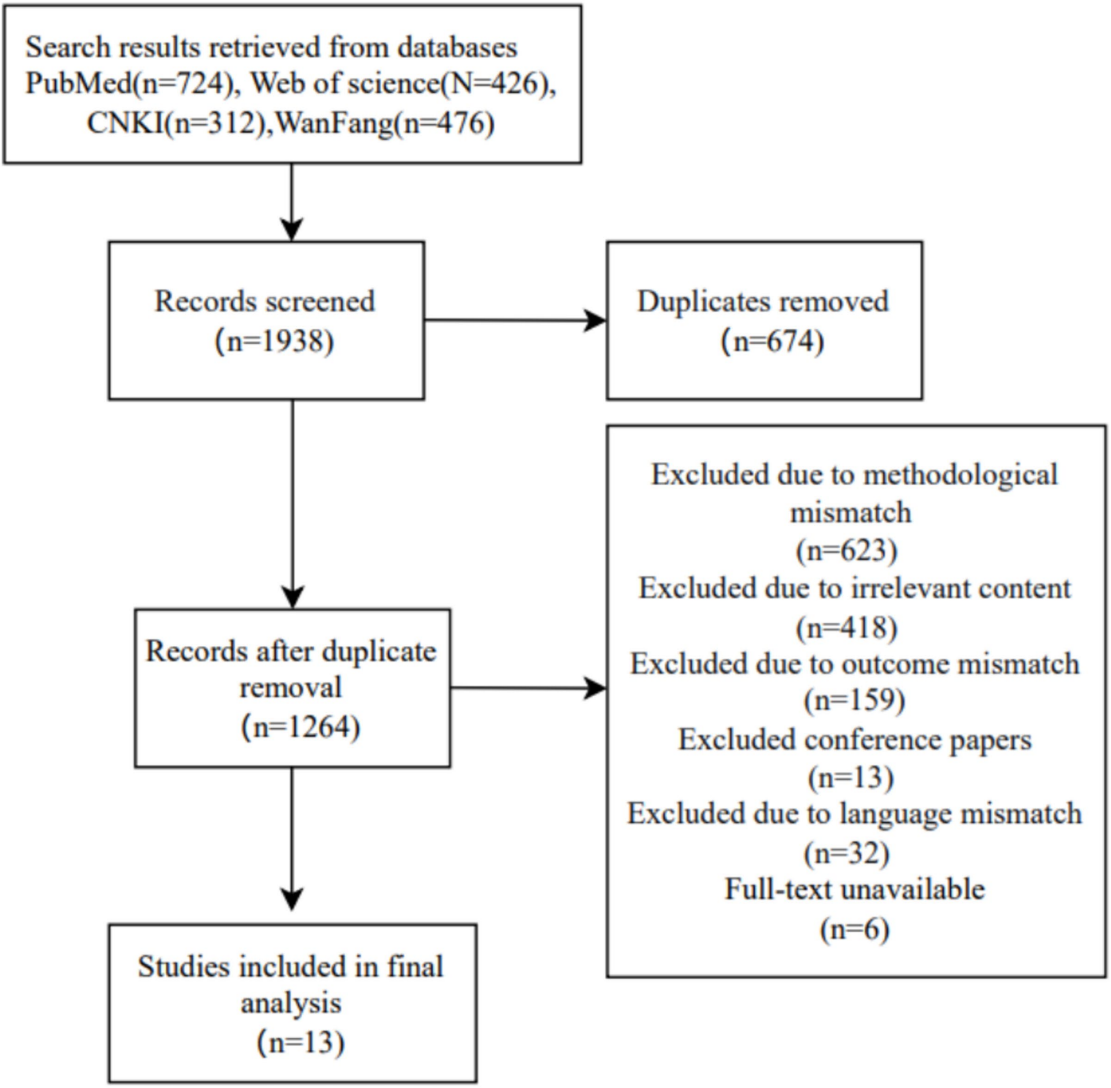
Literature screening flowchart 1.4 Delphi expert consultation.

Based on the thrombosis triad theory and preliminary research findings, the initial risk assessment scale for Deep Vein Thrombosis in plateau areas included 5 first-level indicators and 35 s-level indicators. The literature screening process is illustrated in Figure 1.

#### 2.3.5 Expert selection

According to Delphi method recommendations (15–50 experts), this study invited 22 experts from tertiary hospitals engaged in nursing management and research. Inclusion criteria: (1) bachelor’s degree or higher; (2) intermediate or higher professional title; (3) willingness to participate and complete two rounds of consultation.

#### 2.3.6 Development and distribution of expert consultation questionnaires

The questionnaire consisted of three parts: (1) instructions; (2) importance rating of scale items (5-point Likert scale); (3) expert information survey. The consultation was conducted via email. After the first round, items were revised based on expert feedback. The consultation ended when consensus was reached. Item deletion criteria (15): mean importance score ≤3.5 and coefficient of variation ≥0.25.

### 2.4 Item scoring

Item weights were determined using Saaty’s 1–9 scale through analytic hierarchy process. Scoring rules (16): 1 point for weights 0–0.02; 2 points for 0.02–0.04; 3 points for 0.04–0.06; 4 points for 0.06–0.08; 5 points for 0.08–1.

### 2.5 Pilot testing

Using convenience sampling, 20 patients from Qinghai Provincial Hospital (October 2023) were assessed to evaluate scale clarity and feasibility. Inclusion criteria: (1) ≥ 10 years residence in plateau areas; (2) age ≥18 years; (3) informed consent. Exclusion criteria: (1) communication barriers; (2) impaired consciousness; (3) critical condition within 24 h of admission; (4) severe lower limb trauma preventing ultrasound examination.

### 2.6 Scale item screening and validation

#### 2.6.1 Study participants

A total of 214 inpatients from the Department of Orthopedics, General Surgery, Neurology, and Neurosurgery at Qinghai Provincial Hospital (October–December 2023) were enrolled using convenience sampling, with the same inclusion/exclusion criteria as the pilot test. Ethical approval was obtained (No. 2021-51).

#### 2.6.2 Sample size calculation

The sample size was calculated using MedCalc software (version 23.3.4). The relevant parameters were set as follows: Type I error (Alpha, Significance) = 0.05; Type II error (Beta, 1-Power) = 0.10; Area under the ROC curve = 0.7; Null Hypothesis value = 0.5; Ratio of sample sizes in negative/positive groups = 4.6 (based on a preliminary experiment with 28 subjects, in which 5 developed DVT). The results indicated a required total sample size (both groups together) of 152 subjects, comprising 27 positive cases and 125 negative cases.

#### 2.6.3 Data collection

Data were collected using the developed scale and color Doppler ultrasound. Researchers received standardized training. DVT diagnosis followed the Chinese guidelines for DVT diagnosis and treatment (3rd edition) (17).

#### 2.6.4 Item analysis

(1) Critical ratio method (18): items with scores <3.00 or non-significant differences (*p* < 0.05) between high (top 27%) and low (bottom 27%) scorers were deleted.
(2) Correlation analysis (18): items with correlation coefficients <0.4 or non-significant (*p* < 0.05) with total scores were deleted.
(3) Internal consistency: items whose deletion improved Cronbach’s α were considered for removal.

#### 2.6.5 Validity analysis

(1) Content validity: item-level (I-CVI) and scale-level (S-CVI) content validity indices.
(2) Predictive validity: ROC curve analysis to determine optimal cutoff values.
(3) Discriminant validity: comparing scores between DVT and non-DVT groups.

#### 2.6.6 Reliability analysis

Cronbach’s α coefficients for the overall scale and subdomains, and split-half reliability were calculated.

### 2.7 Statistical analysis

SPSS 26.0 was used. Expert response rate indicated enthusiasm; authority coefficient reflected expertise; Kendall’s coefficient assessed coordination. Significance level was α = 0.05. The ROC curve was generated using R software (version 4.4.1).

## 3 Results

### 3.1 Basic information of experts

A total of 22 experts were finally selected from 6 tertiary hospitals across 3 provinces (Qinghai, Hubei, and Shandong). Among them, 16 experts were from plateau areas and 6 from plain areas. The age range was 28–59 years (42.25 ± 6.45), with working experience ranging from 7 to 40 years (19.60 ± 7.78). Educational background: 13 (59.09%) with bachelor’s degrees, 8 (36.36%) with master’s degrees, and 1 (4.55%) with a doctoral degree. Professional titles: 4 (18.81%) senior, 10 (45.45%) associate senior, 7 (31.82%) intermediate, and 1 (4.55%) junior.

### 3.2 Expert enthusiasm, authority coefficient, and coordination coefficient

The effective questionnaire return rates for the two rounds of expert consultation were 100 and 81.82%, respectively. The authority coefficients were both 0.88. The Kendall’s coordination coefficients were 0.210 and 0.163 (*p* < 0.001).

### 3.3 Indicator modification

Modified indicators: “Previous/current malignant tumors” was simplified to “Malignant tumor” to make the scale more concise; “Surgical history within 1 month (≥45 min)” was changed to “Surgical history within 1 month (≥4 h)” as shorter surgeries pose lower DVT risk. Added indicators: “History of pulmonary embolism” was added due to its strong association with DVT recurrence. “History of lower limb trauma (<1 month)” was added as it increases DVT risk through vascular injury and circulation impairment. Deleted indicators: “Family history of VTE” was removed due to low patient awareness and poor clinical utility. “Antibiotics” was deleted (mean importance score <3.5, CV > 25%, and weak association with DVT risk). “Splenectomy, hepatectomy/pancreatic surgery” was removed due to redundancy with “Major surgery within 1 month.” The preliminary risk assessment scale for Deep Vein Thrombosis in plateau areas included 5 dimensions and 34 items after scoring (Table 1).

**Table 1:** Initial risk assessment scale for deep vein thrombosis in plateau areas.

Primary indicator	Weight	Secondary indicator	Importance score	Coefficient of variation	Weight	Combined weight	Score
General information	0.0864	Age >50 years	4.44 ± 0.62	0.14	0.1549	0.0134	1
		Residence altitude >3,000 m	4.78 ± 0.43	0.09	0.3382	0.0292	2
		BMI > 25 kg/m^2^	4.39 ± 0.5	0.11	0.1087	0.0094	1
		Gender: Female	4.28 ± 0.75	0.18	0.0726	0.0063	1
		Lower limb edema	4.61 ± 0.85	0.18	0.217	0.0187	1
		Smoking history	4.72 ± 0.57	0.12	0.1087	0.0094	1
Medical history	0.2699	Malignant tumor	4.33 ± 1.08	0.25	0.0234	0.0063	1
		History of pulmonary embolism	4.61 ± 0.98	0.21	0.0531	0.0143	1
		History of VTE	4.72 ± 0.67	0.14	0.0951	0.0257	2
		History of lower limb trauma (<1 month)	4.94 ± 0.24	0.05	0.154	0.0416	3
		History of unexplained/habitual abortion (≥3 times)	4.61 ± 0.7	0.15	0.0531	0.0143	1
		History of varicose veins	4.78 ± 0.55	0.11	0.1041	0.0281	2
		Invasive mechanical ventilation	4.67 ± 0.59	0.13	0.0816	0.022	2
		Femoral vein catheterization	4.61 ± 0.7	0.15	0.0531	0.0143	1
		Congestive heart failure/acute myocardial infarction (<1 month)	4.61 ± 0.7	0.15	0.0531	0.0143	1
		Ischemic stroke (<1 month)	4.61 ± 0.7	0.15	0.0531	0.0143	1
		Lower limb arthroplasty	4.67 ± 0.69	0.15	0.0816	0.022	2
		Autoimmune disease	4.39 ± 0.61	0.14	0.0295	0.0079	1
		Severe infection: Sepsis (<1 month)	4.61 ± 0.5	0.11	0.0531	0.0143	1
		Pregnancy/postpartum (<1 month)	4.28 ± 1.07	0.25	0.0209	0.0056	1
		Surgical history within 1 month (≥4 h)	4.56 ± 0.7	0.15	0.0382	0.0103	1
		Immobility/bed rest ≥3 days due to medical condition or doctor’s order	4.61 ± 0.7	0.15	0.0531	0.0143	1
Comorbidities	0.1151	Hypertension	4.33 ± 0.84	0.19	0.1773	0.0204	2
		Diabetes	4.44 ± 0.78	0.18	0.2519	0.029	2
		Hyperlipidemia	4.56 ± 0.78	0.17	0.3591	0.0413	3
		COPD	4.11 ± 0.83	0.2	0.087	0.01	1
		Pulmonary hypertension	4.28 ± 0.75	0.18	0.1246	0.0143	1
Medication history	0.1733	Vasoactive drugs	4 ± 1.08	0.27	0.1095	0.019	1
		Hemostatic drugs	4 ± 1.14	0.28	0.1095	0.019	1
		Anticoagulants	4.5 ± 0.99	0.22	0.2974	0.0515	3
		Current oral contraceptives or estrogen replacement therapy	4.67 ± 0.59	0.13	0.4836	0.0838	5
Laboratory tests	0.3554	Fibrin(ogen) degradation products ≥4.5 mg/L	4.83 ± 0.51	0.11	0.3119	0.1108	5
		Hemoglobin concentration: Female ≥190 g/L, Male ≥210 g/L	4.72 ± 0.46	0.1	0.1976	0.0702	4
		D-dimer ≥0.5 mg/L	4.89 ± 0.32	0.07	0.4905	0.1743	5

### 3.4 Pilot test results

Nursing assessors reported the scale items were clear, easy to understand, and feasible for clinical assessment without operational barriers.

### 3.5 Patient characteristics

Among 214 enrolled patients, 48 (22.43%) developed DVT. Age distribution: <50 years (49 cases, 22.9%) and ≥50 years (165 cases, 77.1%). Gender: male (134 cases, 62.62%) and female (80 cases, 37.38%). Residence altitude: <3,000 m (149 cases, 69.63%) and ≥3,000 m (65 cases, 30.37%).

### 3.6 Item analysis

Following evaluation using the critical ratio method, correlation analysis, and internal consistency testing, no items met all three deletion criteria. For items meeting two deletion criteria, three indicators were removed after group discussion: “smoking history,” “COPD,” and “vasoactive medications.” The rationale for deletion was as follows: “COPD” and “smoking history” are not established common risk factors for DVT and demonstrated weak predictive capability; “vasoactive medications” (including both vasodilators and vasoconstrictors) were removed due to the difficulty in accurately assessing their differential effects on DVT risk in clinical practice, based on considerations of scale utility. The finalized preliminary risk assessment scale for DVT in plateau regions ultimately comprised 5 dimensions and 31 items, as detailed in Supplementary Table S1.

### 3.7 Validity analysis

#### 3.7.1 Content validity

The scale’s I-CVI ranged 0.72–1; S-CVI was 0.91. The “Hemostatic drugs” item had CVI = 0.72 but was retained after expert consultation due to clinical importance. Other items had CVI = 0.78–1.

#### 3.7.2 Predictive validity

**Figure 2:**
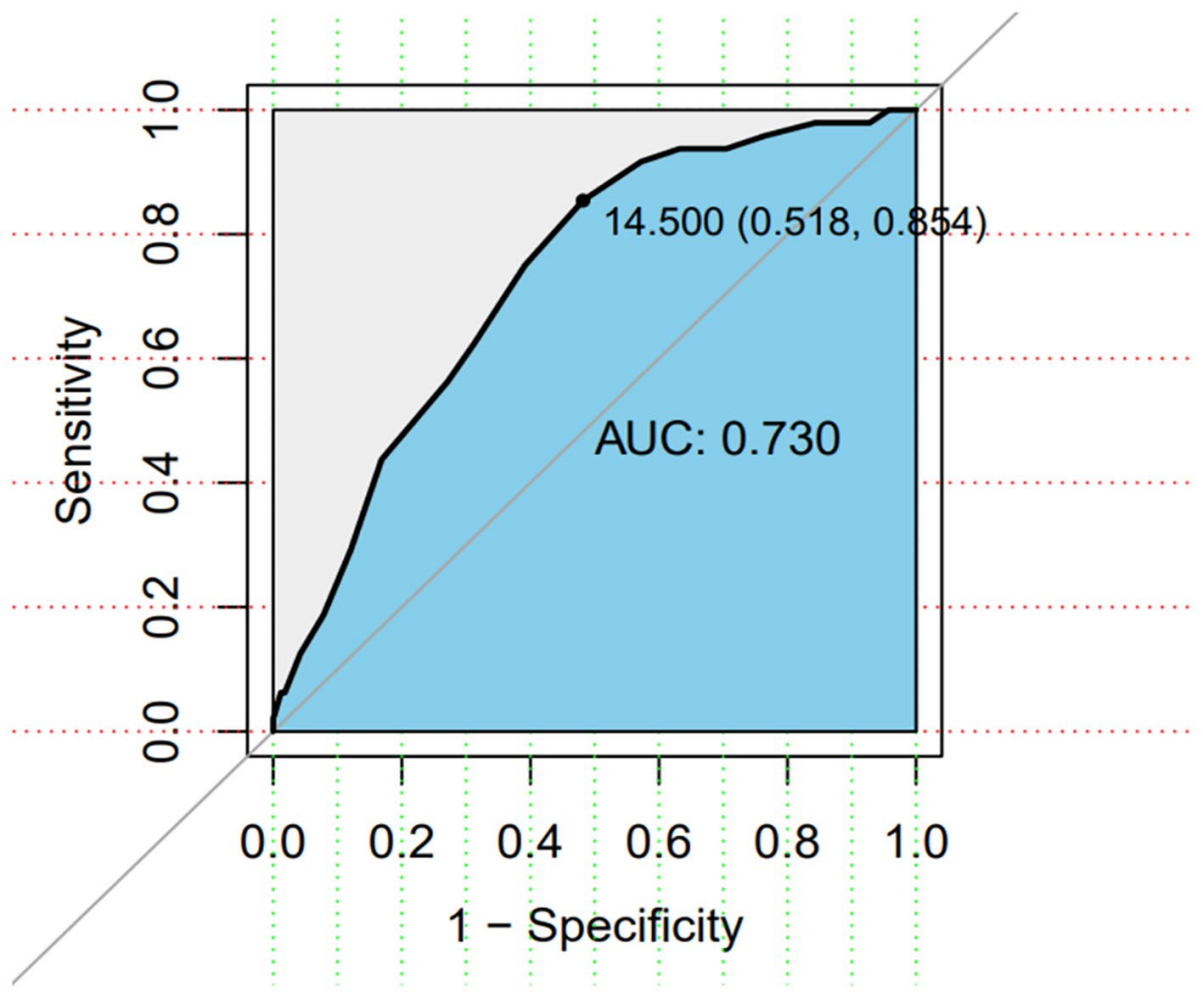
ROC curve of the risk assessment scale for deep vein thrombosis in plateau areas.

The AUC was 0.730 (95%CI:0.655–0.805, *p* < 0.001), indicating good predictive validity (Figure 2). At the optimal cutoff of 14.5 (Youden index = 0.372), sensitivity was 0.854 and specificity was 0.518, positive predictive value was 0.339, and negative predictive value was 0.925.

#### 3.7.3 Discriminant validity and risk stratification

The scale was applied to assess DVT risk in 214 patients. Results showed median (IQR) total scores of 15 (11, 18) for non-DVT group and 18 (16, 20) for DVT group. The non-parametric rank-sum test revealed statistically significant differences (*Z* = −4.514, *p* < 0.001), demonstrating good discriminant validity. Using 15 points as the diagnostic threshold, 42 true positive cases were identified. Risk stratification was established as: low-risk (15–16 points, P0–P25), moderate-risk (17–20 points, P25–P75), and high-risk (≥21 points, P75–P100).

### 3.8 Reliability analysis

The overall Cronbach’s *α* coefficients was 0.953. Subscale Cronbach’s α coefficients: General information: 0.873; Medical history: 0.929; Comorbidities: 0.941; Medication history: 0.778; Laboratory tests: 0.70. The split-half reliability was 0.957, indicating excellent reliability.

## 4 Discussion

### 4.1 The risk assessment scale for deep vein thrombosis in plateau areas demonstrates good scientific validity and reliability

In the preliminary stage of developing this scale, our research team conducted a large-scale, multi-center cross-sectional study that identified key risk factors for DVT in plateau regions (13): age >50 years, residence altitude ≥3,000 m, D-dimer ≥0.5 mg/L, history of varicose veins, current medication use, and comorbidities. These identified risk factors were systematically incorporated as core indicators in the scale development process. Through comprehensive literature review, we ensured broad coverage of DVT risk assessment elements. Compared with commonly used scales like Padua, Autar, Caprini, and Wells, our scale uniquely incorporates “laboratory tests” and “medication history” domains, while encompassing risk factors across multiple clinical departments including orthopedics, ICU, and gynecology, enabling comprehensive risk evaluation. Furthermore, most items of the scale are derived from routinely collected clinical data, ensuring feasibility and efficient implementation even in plateau settings. The Delphi method was strictly followed during expert consultation and item modification, ensuring scientific rigor (19). The scale development involved experts from both plateau (five tertiary hospitals) and plain regions, guaranteeing consideration of regional differences and plateau-specific risk factors, thereby enhancing its applicability. High expert response rates, authority scores, and coordination coefficients further confirm the reliability of our findings (20).

### 4.2 The risk assessment scale for deep vein thrombosis in plateau areas exhibits excellent reliability and validity

The scale developed in this study demonstrated excellent reliability and validity. The overall Cronbach’s *α* coefficient was 0.953, with all subscale coefficients exceeding 0.7, and the split-half reliability reached 0.957, indicating strong internal consistency and high reliability (21). Validity was assessed through content validity, discriminant validity, and predictive validity analyses. The scale’s I-CVI ranged from 0.72 to 1 (S-CVI = 0.91). Although the “hemostatic drugs” item showed a CVI of 0.72, it was retained after expert consultation, while all other items demonstrated CVIs between 0.78 and 1, confirming good content validity and appropriate measurement of target constructs. In the assessment of 214 patients, significant differences in total scores were observed between non-DVT (median = 15, IQR = 11–18) and DVT groups (median = 18, IQR = 16–20), supporting good discriminant validity for distinguishing DVT cases. The ROC curve analysis yielded an AUC of 0.73 (95% CI: 0.655–0.805, *p* < 0.001), indicating satisfactory predictive accuracy (22) (AUC interpretation: 0.5–0.7 = poor, 0.7–0.9 = good, >0.9 = excellent). At the optimal cutoff of 14.5 points (maximum Youden index = 0.372), the scale showed 85.4% sensitivity and 51.8% specificity, correctly identifying 85.4% of DVT cases as high-risk individuals.

### 4.3 The scale demonstrates applicability for plateau regions

This study fully considers the particularity of the plateau environment and the characteristics and pathophysiological features of patients in high-altitude areas. In the “comorbidity” dimension, three high-risk assessment factors for plateau regions were established: “hypertension,” “hyperlipidemia,” and “pulmonary hypertension.” Long-term residence in high-altitude areas leads to hypoxia, which induces vasoconstriction, increased sympathetic nervous system activity, and endothelin release, thereby elevating the risk of hypertension (23, 24). Hypertension, in turn, causes defects in the endothelial nitric oxide system and increased production of endothelium-dependent vasoconstrictors, leading to endothelial dysfunction and a higher risk of thrombosis (25, 26). A meta-analysis indicated that hypertension is associated with an increased incidence of DVT after orthopedic surgery (27). The high-fat, high-calorie, and high-salt dietary habits, along with unique lifestyle patterns in plateau regions, often result in a higher prevalence of hyperlipidemia (28, 29). Hyperlipidemia can disrupt coagulation factors, thereby promoting thrombus formation (30). A cohort study demonstrated that elevated triglyceride (TG) levels are associated with an increased risk of lower extremity DVT (LEDVT) (31). Additionally, hypoxia in high-altitude areas can trigger pulmonary vasoconstriction, increasing pulmonary vascular resistance and inducing pulmonary hypertension (32, 33). Pulmonary hypertension may manifest as increased tissue factor expression, elevated microparticles, and platelet activation, all of which contribute to thrombosis (34). One study revealed that the incidence of DVT in pulmonary hypertension patients (45.83%) (34) was significantly higher than in those with normal pulmonary artery pressure (3.27%) (35). In the “laboratory tests” dimension, the risk assessment factor “hemoglobin concentration: ≥190 g/L for females, ≥210 g/L for males” was established. The threshold for “hemoglobin concentration” was defined according to the “Qinghai Criteria,” the diagnostic standard for chronic mountain sickness established at the Sixth World Congress on Mountain Medicine and High-Altitude Physiology (12). In high-altitude environments, the body gradually increases hemoglobin concentration and hematocrit to enhance oxygen transport capacity in response to chronic hypoxia (36, 37). However, elevated hemoglobin concentration increases blood viscosity, promotes erythrocyte aggregation, and facilitates thrombus formation (38, 39).

### 4.4 Clinical implementation and proposed workflow

For practical application in plateau-area hospitals, we propose that upon admission, nurses should assess all adult inpatients using this scale within the first 24 h, with reassessment following any significant clinical change. The resulting risk category should guide management: low-risk patients (15–16 points) should receive general preventive measures like education, early mobilization, and hydration; moderate-risk patients (17–20 points) should, in addition, be offered mechanical prophylaxis and/or considered for pharmacological prophylaxis after bleeding risk evaluation; and high-risk patients (≥21 points) should receive combined mechanical and pharmacological prophylaxis unless contraindicated, along with consideration for intensified monitoring such as serial ultrasound examinations. This structured approach translates risk stratification into actionable decisions, aiming to optimize resource use and improve DVT prevention in high-altitude settings.

### 4.5 Contribution, limitations, and future directions

The contribution of this study lies in the construction of the first risk assessment scale for deep vein thrombosis specifically targeted at plateau areas through scientific research methods, which incorporates unique risk factors prevalent in high-altitude regions and demonstrates good reliability and validity. However, certain limitations still need to be acknowledged. First, the scale has not yet been validated across different altitude regions. Second, it has not been directly compared with commonly used DVT risk assessment tools. Third, validation was conducted at a single tertiary hospital, which may limit generalizability. Future directions: First, the scale should be compared with existing DVT risk assessment tools to determine its applicability in high-altitude settings. Second, multicenter studies across different altitudes are warranted to validate broader applicability and allow dynamic updates according to changing demographics and comorbidity trends. Third, prospective studies integrating electronic health records can evaluate the impact of the scale on DVT incidence and clinical outcomes. Finally, considering the potential workload of 31 items, a simplified version of the scale could be developed for routine clinical use.

## 5 Summary

Based on a prior large-scale survey and strictly following the Delphi method and analytic hierarchy process, this study developed a high-altitude DVT risk assessment scale comprising 5 dimensions and 31 items. Clinical validation confirmed that the scale exhibits high reliability and validity, demonstrating strong applicability and specificity for high-altitude regions, making it a suitable tool for DVT risk assessment in these areas.

## Data Availability

The raw data supporting the conclusions of this article will be made available by the authors, without undue reservation.
