# Supplementary material for: Construction and validation of the risk assessment scale for deep vein thrombosis in high altitude plateau areas
Source: Front Med (Lausanne). 2026 Mar 11;13:1742559. doi: 10.3389/fmed.2026.1742559 (PMC13013440; doi:10.3389/fmed.2026.1742559)
Supplement: Supplementary file 1 [file Table_1.docx]

Supplementary Table 1:The Risk Assessment Scale for Deep Vein Thrombosis in Plateau Areas

| **The Risk Assessment Scale for Deep Vein Thrombosis in Plateau Areas** | |
| --- | --- |
| General Information | |
| Age >50 years | Yes(1) NO（0） |
| Residence altitude >3000m | Yes(2) NO（0） |
| BMI >25 kg/m² | Yes(1) NO（0） |
| Gender: Female | Yes(1) NO（0） |
| Lower limb edema | Yes(1) NO（0） |
| Medical History | |
| Malignant tumor | Yes(1) NO（0） |
| History of pulmonary embolism | Yes(1) NO（0） |
| History of VTE | Yes(2) NO（0） |
| History of lower limb trauma (<1 month) | Yes(3) NO（0） |
| History of unexplained/habitual abortion (≥3 times) | Yes(1) NO（0） |
| History of varicose veins | Yes(2) NO（0） |
| Invasive mechanical ventilation | Yes(2) NO（0） |
| Femoral vein catheterization | Yes(1) NO（0） |
| Congestive heart failure/Acute myocardial infarction (<1 month) | Yes(1) NO（0） |
| Schemic stroke (<1 month) | Yes(1) NO（0） |
| Lower limb arthroplasty | Yes(2) NO（0） |
| Autoimmune disease | Yes(1) NO（0） |
| Severe infection: Sepsis (<1 month) | Yes(1) NO（0） |
| Pregnancy/Postpartum (<1 month) | Yes(1) NO（0） |
| Surgical history within 1 month (≥4h) | Yes(1) NO（0） |
| Immobility/Bed rest ≥3 days due to medical condition or doctor's order | Yes(1) NO（0） |
| Comorbidities | |
| Hypertension | Yes(2) NO（0） |
| Diabetes | Yes(2) NO（0） |
| Hyperlipidemia | Yes(3) NO（0） |
| Pulmonary hypertension | Yes(1) NO（0） |
| Medication History | |
| Vasoactive drugs | Yes(1) NO（0） |
| Hemostatic drugs | Yes(1) NO（0） |
| Current oral contraceptives or estrogen replacement therapy | Yes(5) NO（0） |
| Laboratory Tests | |
| Fibrin(ogen) degradation products ≥4.5mg/L | Yes(5) NO（0） |
| Hemoglobin concentration: Female ≥190g/L, Male ≥210g/L | Yes(4) NO（0） |
| D-dimer ≥0.5mg/L | Yes(5) NO（0） |
| Total score： low-risk (15-16 points) ; moderate-risk (17-20points ); high-risk (≥21 points) | |
